# How visual experience impacts the internal and external spatial mapping of sensorimotor functions

**DOI:** 10.1038/s41598-017-01158-9

**Published:** 2017-04-21

**Authors:** Virginie Crollen, Geneviève Albouy, Franco Lepore, Olivier Collignon

**Affiliations:** 1 Centre for Mind/Brain Science, University of Trento, Mattarello, Italy; 2 Institute of Psychology (IPSY) and Institute of Neuroscience (IoNS), Université Catholique de Louvain, Louvain-la-Neuve, Belgium; 3 Centre de Recherche en Neuropsychologie et Cognition (CERNEC), Université de Montréal, Montreal, Canada; 4 Movement Control & Neuroplasticity Research Group, Department of Kinesiology, KU Leuven, Belgium

## Abstract

Tactile perception and motor production share the use of internally- and externally-defined coordinates. In order to examine how visual experience affects the internal/external coding of space for touch and movement, early blind (EB) and sighted controls (SC) took part in two experiments. In experiment 1, participants were required to perform a Temporal Order Judgment task (TOJ), either with their hands in parallel or crossed over the body midline. Confirming previous demonstration, crossing the hands led to a significant decrement in performance in SC but did not affect EB. In experiment 2, participants were trained to perform a sequence of five-finger movements. They were tested on their ability to produce, with the same hand but with the keypad turned upside down, the learned (internal) or the mirror (external) sequence. We observed significant transfer of motor sequence knowledge in both EB and SC irrespective of whether the representation of the sequence was internal or external. Together, these results demonstrate that visual experience differentially impacts the automatic weight attributed to internal versus external coordinates depending on task-specific spatial requirements.

## Introduction

Our ability to locate and act on objects in space is a fundamental requirement of our daily life activities. This function is generally achieved by monitoring the position and movement of the body in relation to events located in external space. In touch, spatial localization is initially defined by which receptors on the skin are active; that is, in a skin-based or internal reference frame. However, because the limbs move in the space surrounding the body, the spatial location of an object that enters in contact with the skin and/or on which an action is made requires the brain to integrate internal coordinates with information about current body posture. This process has been referred to as tactile remapping^1,2^ and has been denoted as occurring in external coordinates. The external reference frame refers to a spatial coordinate system that abstracts from the original source but that can still be egocentric – eye-centered, head-centered or trunk centered. “External” should therefore not be understood as implying independence of the body^3,4^. Within this context, tactile localization has been recently defined as a two-step process, in which tactile information (internal coordinates) are first remapped into an external representation^3^. Then anatomical and external spatial information are integrated according to a specific weighting scheme.

The most widespread experimental paradigm that has been used to examine which weighting scheme is associated to the spatial localization of touch is probably the temporal order judgment task (TOJ)^5,6^. In this task, participants have to determine, with their hands uncrossed or crossed over the body midline, which of their two hands received a tactile stimulus first. Crossing the hands actually induces a conflict between the internal and external coordinate systems: with crossed hands, the right hand lies in the left hemispace while the reverse is true for the left hand^5^. Manipulations of posture should not affect performance if spatial localization relied exclusively on internal coordinates. In contrast, if a posture manipulation induces changes in task performance, this would be an indication that the external reference frame has been used to code tactile stimulus location. As sighted adults are strongly impaired in the TOJ task while responding with their hands crossed over the body midline^5,6^, the weighting scheme of touch localization in this population seems to automatically favor, even when it is not necessary, an external reference frame.

In contrast to sighted and late blind individuals, congenitally blind people do not manifest any crossing effects in a static TOJ task^7^. The same observation has interestingly been made whenever congenitally or early blind had to process tactile stimuli^8,9^, the auditory Simon effect^10^, pointing movements toward memorized proprioceptive targets^11^ and even numerical spatial relation^12^. While these findings together suggest that vision drives the development of the automatic integration of internal and external coordinates^5–7,13–16^, recent results demonstrated that bimanual coordination in the congenitally blind was constrained by external-spatial factors, like in the sighted^17^ and that external coordinates may affect tactile localization in congenitally blind in the context of an action that requires external spatial coding (i.e., bimanual arm movements with uncrossed and crossed start and end postures^18^). It is therefore conceivable that congenitally blind do integrate information from internally and externally defined reference frames, but that they do so according to another weighting scheme than the sighted. Integration in the congenitally blind could be restricted to situations in which the use of the non-preferred external reference frame is required by the task^2,18,19^. The experiments reported above suggest that movement is a good candidate to bias spatial localization towards an external coordinate system in sighted as well as in blind individuals. As movements are commonly used to interact with objects located in the external world (e.g., typing on a computer), relying on an external representation within a motor context would indeed seem more appropriate even in early blind individuals.

Interestingly, within the motor literature, a variety of different laboratory-based protocols demonstrated that motor sequence learning involves, like touch perception, the processing of internal and external spatial coordinates. This procedural learning actually refers to the process by which simple, stereotyped movement elements come to be performed effortlessly as a unitary well- rehearsed sequence. In the most classical experiments investigating this process, participants are required to use the fingers of the right or left hand to either press buttons on a keyboard, or to lightly touch one’s own thumb in a precise and sequential order. The sequence of movements may be explicitly^20,21^ or implicitly learned^22^, self-initiated^20^, cued by visual or acoustic stimuli^23^, or interleaved with random movements^24^. But, more importantly for our purposes, the sequence of movements can be learned as both a sequence of finger movements (coded in internal coordinates) and as a sequence of response buttons (coded in external coordinates). These two spatial coordinates can be distinguished by probing skill with the response box turned upside-down (for a review, see ref.^25^). Internal representation of the sequence is assessed by changing the sequence of response locations but preserving the specific pattern of finger movements learned during training; external representation is assessed by changing the specific pattern of finger movements while preserving the sequence of response locations. Despite all the methodological differences of the various motor sequence learning tasks described above, participants typically increase the velocity of their finger movements and decrease the interval between successive key presses with practice, resulting in a decrease of the number of errors made (a measure of accuracy) and in a decrease of the duration necessary to complete the internal and external representations of the learned sequence (a measure of speed). While motor sequence learning has been extensively studied in the sighted population, we still don’t know whether blindness may affect this procedural learning as it affects touch perception.

In this paper, we therefore examined whether vision may differentially shape the use of internal versus external spatial representations of touch and motor sequence learning. Early blind and sighted controls were required to perform 2 tasks. The first task was a tactile Temporal Order Judgment task (TOJ^6^) in which participants had to determine, with their hands uncrossed or crossed over the body midline, which of their two hands received a tactile stimulus first. The second task was a motor sequence learning task^26^. In this task, participants were trained to perform a sequence of five fingers movements. After the training session, participants were tested on their ability to produce, with the same hand but with the keypad turned upside down, the learned (internal condition) or the mirror sequence (external condition). While the TOJ task involves passive touch, action is required to perform the motor sequence learning task. Therefore, if visual deprivation prevents touch localization to be biased towards an external reference frame, but does not prevent the use of external coordinates in the motor sequence learning task, early blind should not manifest any crossing effects in the TOJ task but should be able to produce the internal and external configurations of the learned motor sequence. The use of these tasks on the same participants represents a unique opportunity to test the idea that early blind do integrate information from different reference frames, but use the external coordinates with a higher selectivity according to the requirements of the task, therefore using another weighting scheme than the sighted^17,18^.

## Method

### Participants

Eleven blind participants and 11 sighted controls (SC) took part in the study. The SC and blind groups were matched in terms of age, sex and musical knowledge (i.e., number of practices a week). The blind group was composed of 3 females and 8 males ranging in age from 21 to 61 years old with a mean age of 42 years (*SD* = 13.74). Nine participants were right handed, 2 were ambidextrous. Causes of blindness included detachment of the retina, congenital cataract, optic nerve burned, retinitis pigmentosa, congenital malformation, retinoblastoma, medical accident, thalidomide, retinopathy of prematurity and Leber’s congenital amaurosis. Nine participants were congenitally blind (CB), two were early blind (EB). One lost his sight at 2 months, and one lost vision in the left eye at 10 months and vision in the right eye at 3 years. In this group (that we will call thereafter the EB group), 7 participants had musical training. Blind individuals were totally blind or had only rudimentary sensitivity for brightness differences but never experienced patterned vision. The SC group was composed of 4 females and 7 males ranging in age from 21 to 68 years old with a mean age of 43 years (*SD* = 14.13). As in the EB group, 7 participants of the SC group were trained musicians (4 of them were professional musicians). Musical abilities of both groups were matched as it has already been demonstrated that musicians show greater efficiency than non-musicians in motor sequence learning task^27^. Sighted participants were blindfolded when performing the tasks. The samples size was determined by the number of blind participants we were able to recruit on a 6 months period. A minimal number of 10 participants was used as a cut-off since previous studies^7^ have shown reliable results with such a number. All the procedures were approved by the Research Ethics Boards of the University of Montreal. All experiments were performed in accordance with relevant guidelines and regulations and informed consent was obtained from all participants. Below, we report all experimental manipulations, all exclusions of data (if any), and all evaluated measures of the study.

### Tasks

#### Temporal order judgment task

In this task, we used a similar procedure as the one applied by Röder and collaborators^7^. Two successive tactile stimuli were presented for 10 ms to the distal phalanxes of the left and right middle fingers at 10 different stimulus onset asynchronies (SOAs): −200, −90, −55, −30, −15, 15, 30, 55, 90, 200. Negative values indicated that the first stimulus was presented to the participant’s left hand; positive values indicated that the first stimulus was presented to the participant’s right hand. Tactile stimuli were delivered using a pneumatic tactile stimulator (Institute for Biomagnetism and Biosignal Analysis, University of Muenster, Germany). A plastic membrane (1 cm in diameter) was attached to the distal phalanxes of the left and right middle fingers and was inflated by a pulse of air pressure delivered through a rigid plastic tube. The plastic tube connecting the stimulator to the participants’ finger tips were inserted into the testing room through a hole padded with sound attenuating foam to ensure that tactile stimulations were completely silent from the inside of the room. Participants had to press a response button placed below the index finger of the hand that they perceived to have been stimulated first. Participants were asked to perform the task either with their hands in a parallel posture (i.e., uncrossed posture) or with their arms crossed over the body midline. The order of posture conditions was counterbalanced across participants. Hand posture was altered every two blocks. Participants had to respond within a random interval ranging from 3000 to 4000 ms (from the onset of the target) otherwise the trial was terminated. Each SOA was presented 32 times in both hand postures, giving rise to 640 trials in total. These 640 trials were presented through 8 blocks of 80 stimuli. Prior to the experiment, participants had to complete two blocks of 16 practice trials (one block in the uncrossed posture followed by one block in the crossed posture). Stimuli were delivered and reaction times were recorded using Presentation software (Neurobehavioral Systems Inc.) running on a Dell XPS computer using a Windows XP operating system. The two response keys were placed 40 cm in front of the participant’s body and 50 cm away from each other. During testing, participants sat in a silent room with the head restrained by a chin rest. Participants also wore earplugs to mask any sounds made by the operation of the tactile stimulators.

### Motor sequence learning task

**Figure 1:**
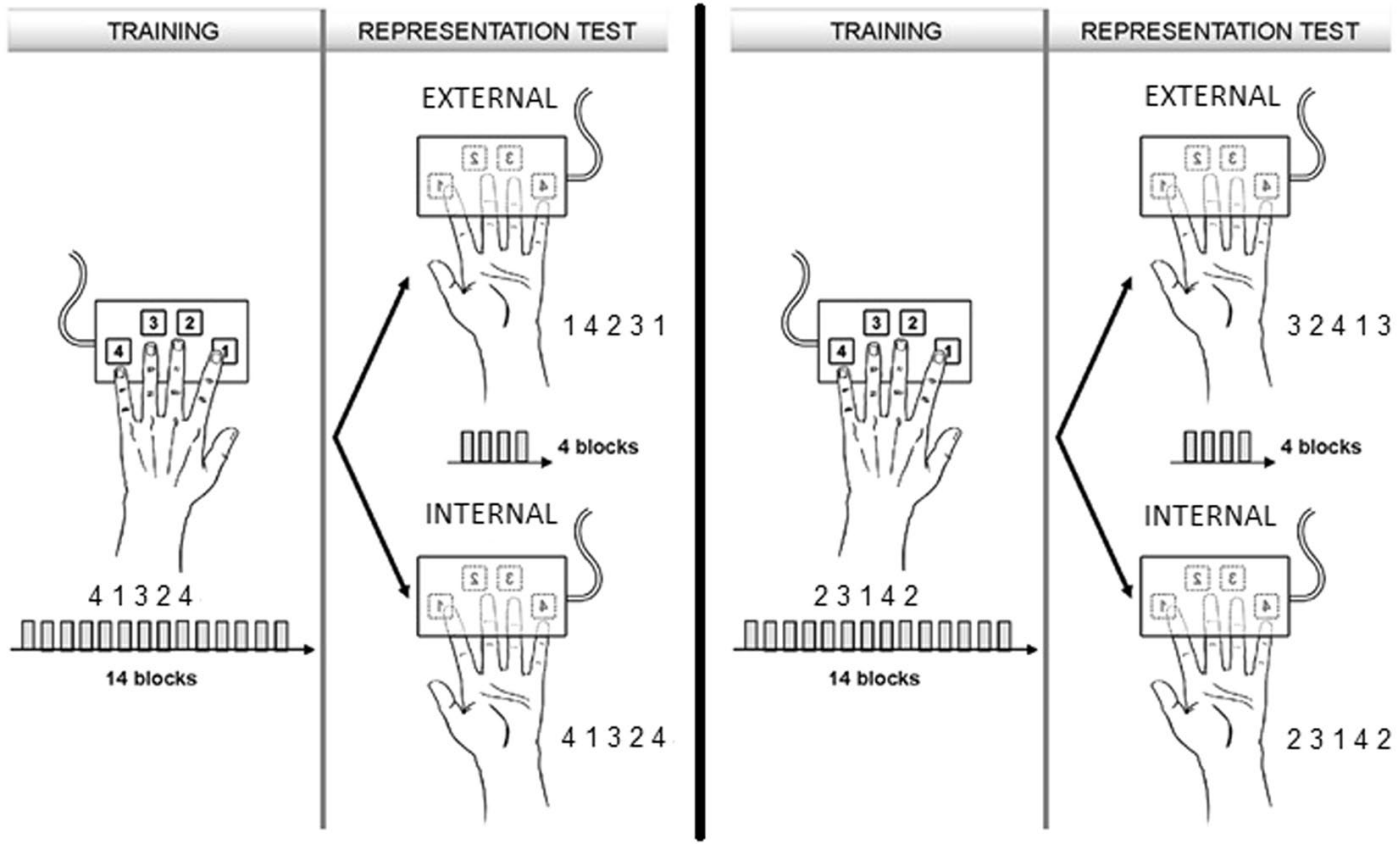
Motor sequence learning task. Training panel: All the participants were trained with the usual set-up (hand on the keypad). Representation test panel: immediately after initial training, switching the keypad and hand coordinates upside down allowed us to investigate the EXTERNAL (same spatial sequence but different finger movements) and INTERNAL (same finger movements but different spatial sequence) representations. Participants were tested on both representations with two different motor sequences: 41324 (left side of the figure) vs. 23142 (right side of the figure).

This task involved 2 separate practice sessions referred to as the training and the representation test sessions. On each period, participants had to tap on a keyboard, with their non-dominant hand, a five-element finger sequence as rapidly and as accurately as possible. The sequence to perform was explicitly told to the participants before training. The sequence was performed in 14 successive practice blocks during the training session and 4 successive blocks during the representation test session, each practice block (composed of 60 key presses, a maximum of 12 repetitions of the same sequence) being separated by 15-second rest intervals (Fig. 1). During the training period, participants had to perform the sequence in a usual set-up (i.e., with the non-dominant hand on the keyboard and the keyboard upside-up). Two different sequences were used to allow the investigation of the internal and external representations within subject. As a within-subject design was used and in order to avoid between-sessions retention of motor performance, participants were trained on a different sequence at each occasion (either 4 1 3 2 4 or 2 3 1 4 2, where 1 corresponds to the index finger and 4 to the little finger, see Fig. 1). These 2 sequences had the same level of complexity: the length of the 2 sequences (41324 vs. 223142) was the same (i.e., 5 elements) and the rule defining the architecture of the sequences was identical (i.e., 3 fingers pressed once and one finger pressed twice in a sequence) (see also supplemental data). During the representation test session, participants had to perform the sequence with the hand and the keyboard turned upside-down. The presence of external and internal representations of the sequence was assessed in this session at an individual level. The external representation was assessed by changing the finger movements that participants needed to perform, while preserving the spatial locations of the response buttons on the reverted key-pad (from sequence 4 1 3 2 4/2 3 1 4 2 to their mirror configurations 1 4 2 3 1/3 2 4 1 3, respectively). The internal representation of the sequence was in contrast assessed by changing the spatial locations of the response buttons while preserving the order of finger movements learned during training (i.e., sequence 4 1 3 2 4 or 2 3 1 4 2, see Fig. 1). All participants were tested on both representations in approximately one week of interval. The order of representation tested (external or internal) and the sequences used were counterbalanced across participants. On session 1, each participant practiced one of the two sequences (41324 or 23142) and was subsequently tested on one of the two transfer conditions (either external or internal). On session 2, each participant practiced the other sequence and was tested on the other of the two transfer conditions. In both sessions, the transfer test took place immediately (i.e., less than 5 min) after initial training. Motor skill performance was measured in terms of speed (block duration to perform the 60 key presses) and accuracy (number of accurate sequences by block). The task was implemented in MATLAB (Mathworks Inc., Sherbom, MA) using the Cogent 2000 toolbox (http://www.vislab.ucl.ac.uk/cogent.php).

### Procedure

Participants were tested in three different sessions: they performed the 2 conditions of the motor sequence learning task in the first 2 sessions (separated by approximately one week of interval), then they performed a third session (one month later) during which the TOJ task was presented. The motor sequence learning task was split over two sessions to avoid any interfering effect between motor practice sessions. A window of ⟪sensitivity⟫ to interference in the first 4–6 hours following initial training has indeed been described in the literature^28,29^.

### Data analysis

#### Temporal order judgment task

The mean percentages of “right hand first” responses were first calculated for each participant, SOA and posture. These raw proportions were transformed into their standardized z-score equivalents and then used to calculate the best-fitting linear regression lines of each participant^5^. Because the longest intervals showed evidence of a ceiling effect for the uncrossed posture, only the intermediate 8 points (i.e., −90 to 90 ms) were included in the analysis. The slopes of each individual line were then submitted to an ANOVA with posture (uncrossed vs. crossed) as the within-subject factor and group (EB, SC) as the between-subject variable.

The just noticeable difference (JND; the smallest interval needed to reliably indicate temporal order) was secondly calculated from the mean slope data by subtracting the SOA needed to achieve 75% performance from that needed to achieve 25% performance and dividing by two^2^. This value could not be determined independently for all observers because several of them obtained a slightly negative slope value for the crossed posture; indicating that these participants often responded with the opposite hand as the one that has been stimulated first^5^.

#### Motor sequence learning task

In the motor sequence learning task, we first evaluated whether practice of the sequence in the training session improves participants’ performance. A 14 (blocks of practice during the training session) × 2 (condition: external vs. internal) × 2 (group: EB, SC) ANOVA was therefore conducted on speed of performance (i.e., block duration in ms) and accuracy (i.e., number of correct sequences per block). We also examined participants’ performance in the representation session by conducting a 4 (blocks of practice during the representation session) × 2 (condition: external vs. internal) × 2 (group: EB, SC) ANOVA on speed of performance and accuracy.

Finally, to examine the transfer in sequence knowledge, taken as an indicator of the development of external and internal representations, the averaged performance of the first four blocks of training was compared to the four blocks of the representation test session. We tested this with a three-way ANOVA with the averaged performance of the first four blocks of training and the four blocks of the representation session as the first within-subject factor (session), the type of representation (external vs. internal) as the second within subject factor and the group (EB, SC) as the between-subject factor. As previously, this analysis was performed on speed of performance and accuracy. Comparing the beginning of the training session (first 4 blocks) to the representation session (4 blocks) is a procedure generally used to assess the amplitude of the transfer of sequence knowledge^26,30^.

## Results

### Temporal order judgment task

**Figure 2:**
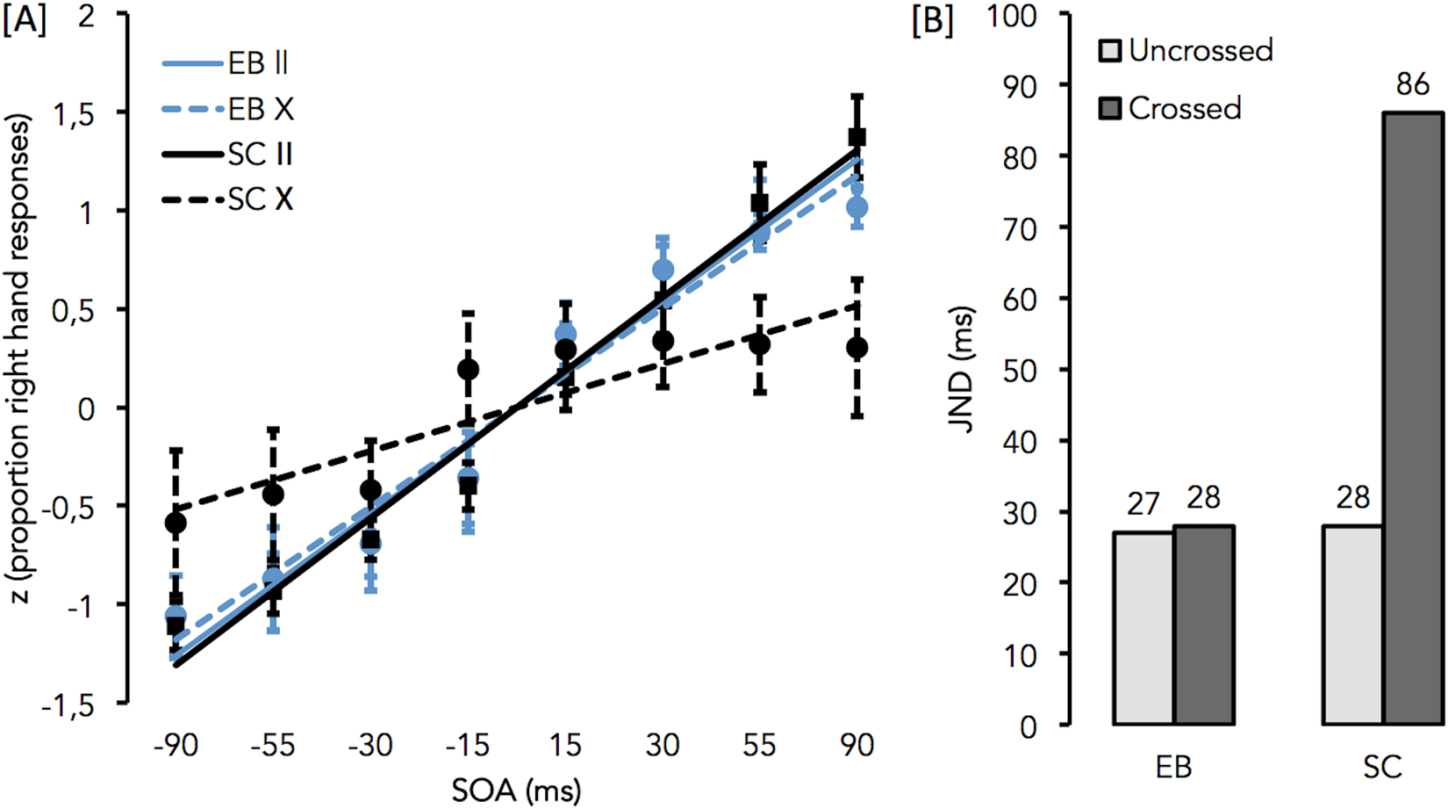
(**A**) Standardized z-score equivalents of the mean proportions of right-hand responses and best-fitting linear regression lines for the uncrossed (II – continuous lines) and crossed (X - dotted lines) postures for EB (in blue) and SC (in black). Bars represent standard error of the mean; (**B**) JND: the minimum interval between the two tactile stimuli required for participants to judge their temporal order accurately on 75% of trials. No error bars are presented as the JND was calculated on the group data.

Results of the 2 (postures: uncrossed vs. crossed) × 2 (groups: EB, SC) ANOVA carried out on the slopes of each individual regression lines showed: (1) a significant effect of posture, *F*(1, 20) = 13.93, *p* = 0.001, *η* ^*2*^ = 0.41, the regression line for the uncrossed posture being steeper (*M* = 0.90 ± 0.03) than the regression line for the crossed posture (*M* = 0.59 ± 0.09); (2) a significant effect of group, *F*(1, 20) = 8.47, *p* = 0.009, *η* ^*2*^ = 0.30, the EB (*M* = 0.90 ± 0.08) performing better than the SC (*M* = 0.59 ± 0.08); and (3) a significant posture × group interaction, *F*(1, 20) = 11.37, *p* = 0.003, *η* ^*2*^ = 0.36. To further examine this interaction, paired samples t-test were performed on each group separately with hand position as the only factor. In SC group, participants’ performance was better in the uncrossed posture (*M* = 0.88 ± 0.06) than in the crossed posture (M = 0.29 ± 0.18), *t*(10) = −3.61, *p* = 0.005 (see Fig. 2A). In deep contrast, the performance did not significantly differ between hand postures in the EB group, *t*(10) = −0.98, *p* = 0.350, the slope of the regression lines being similar in the uncrossed (*M* = 0.91 ± 0.01) and crossed postures (*M* = 0.88 ± 0.04). As the development of spatial representation is sensitive to early visual deprivation during the first years/months of life^31^, we performed the same analysis without the 2 EB participants. Only the 9 congenitally blind were included. This analysis yielded the same results as the one described above (see supplemental material for a detailed description of the results).

Moreover and as shown in Fig. 2B, crossing the hands led to a significant decrement in performance in SC; it actually more than doubled the JND. The EB group, in striking contrast, was not affected by the crossing of their hands as already reported by Röder *et al*.^7^. Since both groups of participants presented similar level of performance in the uncrossed position, this observation could not be explained by better temporal resolution ability in EB.

### 3.2 Motor sequence learning task

#### Training session

**Figure 3:**
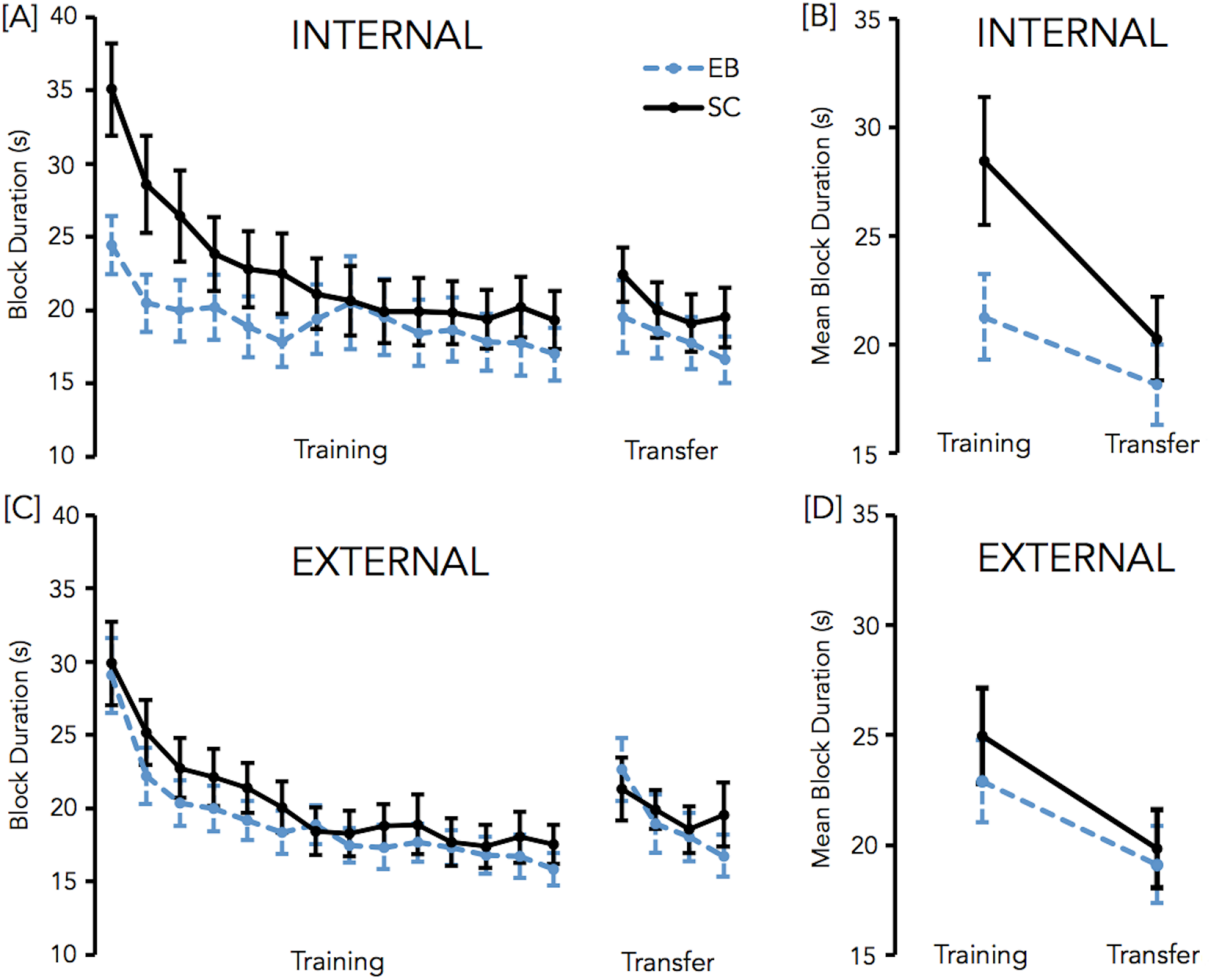
Results of the motor sequence learning task. Mean block duration during the 14 blocks of the training and the 4 blocks of the representation test (transfer) sessions for the internal (**A**) and external conditions (**C**) of the task. Transfer of sequence knowledge in the internal (**B**) and external (**D**) conditions of the task. Bars represent standard error of the mean.

The 14 (blocks of practice during the training session) × 2 (representation: external vs. internal) repeated measures ANOVA conducted on speed of performance (i.e., block duration in ms), with group (EB, SC) as the between-subject factor, yielded a significant main effect of block, *F*(13, 260) = 49.80, *p* = 0.000, *η* ^*2*^ = 0.71, indicating that block duration decreased with practice. There was also a significant block × group interaction, *F*(13, 260) = 3.77, *p* = 0.02, *η* ^*2*^ = 0.16, indicating that the learning curve (changes in performance from one block to the other) differed between SC and EB. Data inspection revealed that the learning curve was steeper in SC due to slower performance at the beginning of training. A significant block × group × representation interaction, *F*(13, 260) = 3.01, *p* = 0.043, *η* ^*2*^ = 0.13 was also observed. However, follow-up analyses indicated no block × representation interaction within each group (all *F* < 3.16, all p-values > 0.06). No other effect was significant (see Fig. 3).

The same 14 (blocks of practice during the training session) × 2 (condition: external vs. internal) × 2 (group: EB, SC) ANOVA conducted this time on the accuracy scores (number of accurate sequences per block) did not reveal any significant effect (all *p*_*s*_ > 0.05).

*Representation test sessions*. The 4 (blocks of practice in the representation test session) × 2 (representations: external vs. internal) repeated measures ANOVA carried out on performance speed with group (EB, SC) as the between-subject factor revealed a significant main effect of block, *F*(3, 60) = 24.81, *p* = 0.000, *η* ^*2*^ = 0.55, block duration decreasing with practice for the two representations of the sequence (see Fig. 3). No other effect was observed.

The same 4 (blocks of practice in the representation test session) × 2 (representations: external vs. internal) × 2 (groups: EB, SC) ANOVA conducted on the accuracy measure did not show any significant effect, accuracy remaining stable with a low error rate throughout the 4 blocks of practice, *F*(3, 60) = 1.06, *p* = 0.395, *η* ^*2*^ = 0.05, whatever the representation tested, *F*(1, 20) = 0.21, *p* = 0.651, *η* ^*2*^ = 0.01, and in the 2 groups of participants, *F*(1, 20) = 0.05, *p* = 0.817, *η* ^*2*^ = 0.003.

*Transfer between the training and the representation test sessions*. The 2 (session: first 4 blocks vs. last 4 blocks) × 2 (representation: external vs. internal) × 2 (group: EB, SC) ANOVA performed on block duration revealed a significant main effect of session, *F*(1, 20) = 35.16, *p* = 0.000, *η* ^*2*^ = 0.64, showing an improvement of performance from the training to the representation test session (see Fig. 3). No between-groups differences, *F*(1, 20) = 1.46, *p* = 0.241, *η* ^*2*^ = 0.07, and no interactions were observed. Interestingly, the representation × session × group interaction was not significant, *F*(1, 20) = 1.76, *p* = 0.199, *η* ^*2*^ = 0.08, showing that EB and SC both demonstrated significant transfer of motor sequence knowledge irrespective of whether the representation of the sequence was external or internal. The same analysis performed without the 2 early blind participants yielded the same results as the one described above (see supplemental material for a detailed description of the results). Moreover, to further examine the absence of the representation × session × group interaction on block duration measures, we computed Bayesian statistics with JASP^32^. These statistics have the main advantages of quantifying evidence instead of forcing an all-or-none decision. Bayes factors indeed provide a coherent approach to determining whether non-significant results support a null hypothesis (interaction absent) over a theory (interaction present), or whether the data are just insensitive. This analysis highlighted a BF_01_ of 4.65, indicating that the posterior probabilities were 0.82 for H_0_ (the null hypothesis has 82% chance of being true) and 0.18 for H_1_. According to Raftery’s (1995)^33^ classification of evidence into weak (0.50–0.75), positive (0.75–0.95), strong (0.95–0.99), and very strong (>0.99), the probability value obtained here provided positive support for H_0_.

A 2 (session: first 4 blocks vs. last 4 blocks) × 2 (representation: external vs. internal) × 2 (group: EB, SC) ANOVA was finally performed on accuracy scores. It only revealed a significant effect of session, *F*(1, 20) = 6.51, *p* = 0.019, *η* ^*2*^ = 0.55, indicating improvement in performance accuracy. No other results were significant.

## Discussion

We aimed to investigate the role visual experience plays in shaping the use of internal and external coordinate systems for sensori-motor processing. The same participants were involved in two different tasks allowing us to directly compare their use of different spatial coordinates in the sensory and motor fields. The spatial representation of touch was assessed by asking participants to perform a TOJ task with the hands uncrossed or crossed over the body midline. The spatial representation of motor plans was tested by requiring participants to reproduce a motor sequence with the hand turned upside-down either following internal or external coordinates.

Results of the TOJ task replicated the data of Röder *et al*.^7^: in SC, crossing the hands reduced the slope of the regression line while no decrease of performance was observed in EB. This absence of crossed-hand effect in CB and EB is attributed to a difference in the weights that are used to integrate internal and external spatial information. While the weighting scheme of SC automatically favors an external coordinate system, EB preferentially rely on an internal frame of reference to perform the task. The automatic integration of internal and external coordinates for touch localization therefore appears to be driven by developmental vision. Such integration probably helps the alignment of the spatial frames of references that are used by the distal senses (e.g., vision and audition) and the body limbs. For example, our ability to interact with our immediate surroundings depends on our ability to represent the location of objects with respect to our own body and especially to our hands. This process is particularly critical since the hands move constantly within the space around our body as different postures are adopted. It has therefore been demonstrated that EB have more difficulties to optimally integrate audio-tactile information in the crossed posture due to the poorly aligned spatial coordinates of these two modalities in such conditions^9^. However, since the TOJ task may be resolved using skin-based coordinates only, the weighting scheme used by the EB shields them from the detrimental crossing effect. Interestingly, a similar difference in the weights that are used to integrate anatomical skin-based and external spatial information has been observed in patient HS, a man who had been totally deprived of vision for the first two years of life^33^. This suggests the presence of a sensitive period early in life for the development of the automatic use of an external visuo-spatial frame of reference for coding touch in space^34^. It is therefore possible that early visual deprivation alleviates the weights of external coordinates due to the reorganization or to the lack of development of the brain circuits implicated in such process. The posterior parietal cortex (PPC) has been hypothesized to play a crucial role in implementing such operation in SC^35^ and this region has been repetitively shown reorganized in EB^36–39^. The role of the PPC in touch localization is partially supported by an electroencephalographic study showing that the detection of deviant tactile stimuli at the hand induced event-related potentials that varied in crossed when compared to uncrossed condition of posture in sighted subjects, whereas changing the posture of the hand had no influence on the early blind subjects’ brain activity^40,41^.

Motor sequence learning, on the other hand, has been shown to encompass two independent processes named “spatial” (external) and “motor” (internal)^42–44^. Within this view, learning a piano sonata not only requires performing specific series of finger movements (in an internal reference frame) but also requires learning the position of specific musical notes in an external reference frame. In our second experiment, a motor sequence-learning task was used to characterize the effect of visual experience on the creation of both external and internal motor representations. The existence of these two representations after an initial learning phase was measured using a “transfer” protocol in which all participants were tested on their ability to produce the internal or external-spatial sequence with the same hand, but with the keypad turned upside down (see Fig. 1). By reversing the keypad, the same finger movements were no longer associated with the identical spatial sequence in external space and vice versa^26^. Accordingly, such a manipulation generated two different sequence representations: an internal representation that probed movement-based learning and an external representation that probed external spatial learning^26^. As expected, we observed that SC developed both external and internal representations of the sequence. Blocks’ duration indeed decreased from the training to the representation test session in the external as well as in the internal conditions of the study. Crucially, our results show that EB were similarly able to develop these two spatial representations. As an absence of evidence that group differences exist is not necessarily an evidence for the true absence of such difference, one may suggest that our motor sequence learning task was not sensitive enough to highlight true differences in spatial representation between the two groups. We however do not believe this hypothesis is the most parsimonious. First, our complementary Bayesian statistics support the idea of a “true” absence of group differences. Second, the observation that both groups of participants were able to implicitly create an internal and an external spatial representation of their motor action parallels previous studies also showing an absence of difference between the sighted and blind groups to support the idea that vision is not necessary to the development of external coordinates in motor coordination^17^.

While the effector-dependent representation is supported by a striato-motor network^45–48^, the effector-independent motor representation has been found to recruit an hippocampo-cortical network^35^ involving prefrontal and parietal cortices^42–44,46^. Even if visual inputs are the predominant sensory inputs of the parietal cortex, auditory and somatosensory information also access this area^49,50^. Makin, Holmes and Zohary^51^ demonstrated that the posterior intraparietal sulcus (IPS) and lateral occipital complex represent hand-centered space in a predominantly visual manner, whereas the anterior IPS was characterized by a more proprioceptive representation of the space surrounding the hand. It is therefore possible that EB mainly rely on the anterior IPS to code an external representation of the space surrounding the hand. Through the proprioceptive and auditory modalities, EB people might therefore localize objects in the external space and produce a goal-directed action toward them. Such non-visual sensory-motor loop may be sufficient to build an external sense of space, which is used to act in the external environment. In support of this hypothesis, it was demonstrated that the parieto-occipital reach-related regions retain their functional role — encoding of the spatial position of the reach target — in EB^52^.

The fact that SC and CB performed differently in the TOJ task could be explained by the idea that CB, in contrast to SC, do not integrate internal and external spatial information by default. However, the fact that CB and SC behaved similarly in the motor sequence learning task is more in accordance with the idea that both groups integrate spatial information from different reference frames but do this integration according to different weighting schemes^4,18,19^. While integration seems automatic in SC, external coordinates are used by the CB when the focus of the task is on external coordinates (as in the external condition of the motor sequence learning task). To summarize, our results therefore suggest task-specific differences in the way blind and sighted use specific spatial frame of references for sensori-motor processing^4^. It has been argued that tactile localization is a two-step process, in which tactile information is first remapped to an external representation^4^. Then anatomical and external spatial information are integrated. The weights used for integration are presumably determined by early visual experiences and by the current task demands. While external coordinates are more weighted in the SC while they perform the TOJ experiment, this does not prevent the external representation of motor action in EB. In other words, our data do not support the idea that early visual experience is necessary for the development of an external coordinate system for perception and action. Our results rather suggest that, even if such external frame of reference is less automatically activated in early blind for the processing of touch, it is readily accessible when participants have to perform an action in the external world^17,18^.

## Electronic supplementary material

Supplementary info
