# Author Correction: How visual experience impacts the internal and external spatial mapping of sensorimotor functions

**DOI:** 10.1038/s41598-017-16992-0

**Published:** 2017-12-04

**Authors:** Virginie Crollen, Geneviève Albouy, Franco Lepore, Olivier Collignon

**Affiliations:** 1 Centre for Mind/Brain Science, University of Trento, Mattarello, Italy; 2 Institute of Psychology (IPSY) and Institute of Neuroscience (IoNS), Université Catholique de Louvain, Louvain-la-Neuve, Belgium; 3 Centre de Recherche en Neuropsychologie et Cognition (CERNEC), Université de Montréal, Montreal, Canada; 4 Movement Control & Neuroplasticity Research Group, Department of Kinesiology, KU Leuven, Belgium

*Scientific Reports* **7**:1022; doi:10.1038/s41598-017-01158-9; Article published online 21 April 2017

The original version of this Article listed incorrect affiliations for Virginie Crollen and Olivier Collignon. The correct affiliations are listed below:

Virginie Crollen:

Centre for Mind/Brain Science, University of Trento, Mattarello, Italy.

Olivier Collignon:

Centre for Mind/Brain Science, University of Trento, Mattarello, Italy.

Institute of Psychology (IPSY) and Institute of Neuroscience (IoNS), Université Catholique de Louvain, Louvain-la-Neuve, Belgium.

In addition, the author contributions statement was incomplete, where

“G.A. designed research; V.C. performed research; V.C., G.A. and O.C. analyzed data; V.C., G.A., F.L., and O.C. wrote the paper”.

now reads:

“G.A., V.C. and O.C designed research; V.C. performed research; V.C., G.A. and O.C. analyzed data; V.C., G.A., F.L., and O.C. wrote the paper”.

These errors have been corrected in the HTML and PDF versions of this Article.

