# Supplementary material for: How visual experience impacts the internal and external spatial mapping of sensorimotor functions
Source: Sci Rep. 2017 Apr 21;7:1022. doi: 10.1038/s41598-017-01158-9 (PMC5430802; doi:10.1038/s41598-017-01158-9)
Supplement: Supplementary file 1 — Supplementary info [file 41598_2017_1158_MOESM1_ESM.doc]

**How visual experience impacts the internal and external spatial mapping of sensorimotor functions**

Virginie Crollen, Geneviève Albouy, Franco Lepore and Olivier Collignon

**Supplemental data**

*Temporal order judgment task*

A 2 (postures: uncrossed vs. crossed) x 2 (groups: EB, SC) ANOVA including the 9 congenitally blind only and carried out on the slopes of the individual regression lines showed: (1) a significant effect of posture, *F*(1, 18) = 10.62, *p* = .004, *η^2^* = .37; (2) a significant effect of group, *F*(1, 18) = 8.15, *p* = .011, *η^2^* = .31; (3) a significant posture x group interaction, *F*(1, 18) = 10.45, *p* = .005, *η^2^* = .37. These results are therefore similar to the one reported in the main text of the manuscript. It suggests that the inclusion of the 2 EB in the main analyses do not influence the conclusions of our paper (see supplemental Figure 1 for a representation of individual data in the blind group)

*Complexity of the 2 sequences used in the motor sequence learning task*

To evaluate whether the 2 sequences had the same level of complexity, we carried out a 14 (blocks of practice in the training session) x 2 (sequences: 41324 vs. 223142) repeated measures ANOVA on participants’ RT. The sequence effect was not significant, F(1, 42) = 0.21, p > .6, η^2^ = .005, and there was no blocks x sequence interaction, F(13, 546) = 1.93, p > .1, η^2^ = .04, thus suggesting similar level of performance between the 2 sequences.

*Transfer between the training and the representation test sessions of the motor sequence learning task.* A 2 (session: first 4 blocks vs. last 4 blocks) x 2 (representation: external vs. internal) x 2 (group: blind, sighted) ANOVA only including the 9 congenitally blind (CB) participants was performed on the block duration data. It revealed a significant main effect of session, *F*(1, 18) = 28.56, *p* = .000, *η^2^* = .61, showing an improvement of performance from the training to the representation test session. No other effect was significant. These results are therefore similar to the one reported in the main text. It suggests that the inclusion of the 2 EB in the main analyses do not influence the conclusions of our paper (see supplemental Figure 1 for a representation of individual data in the blind group).


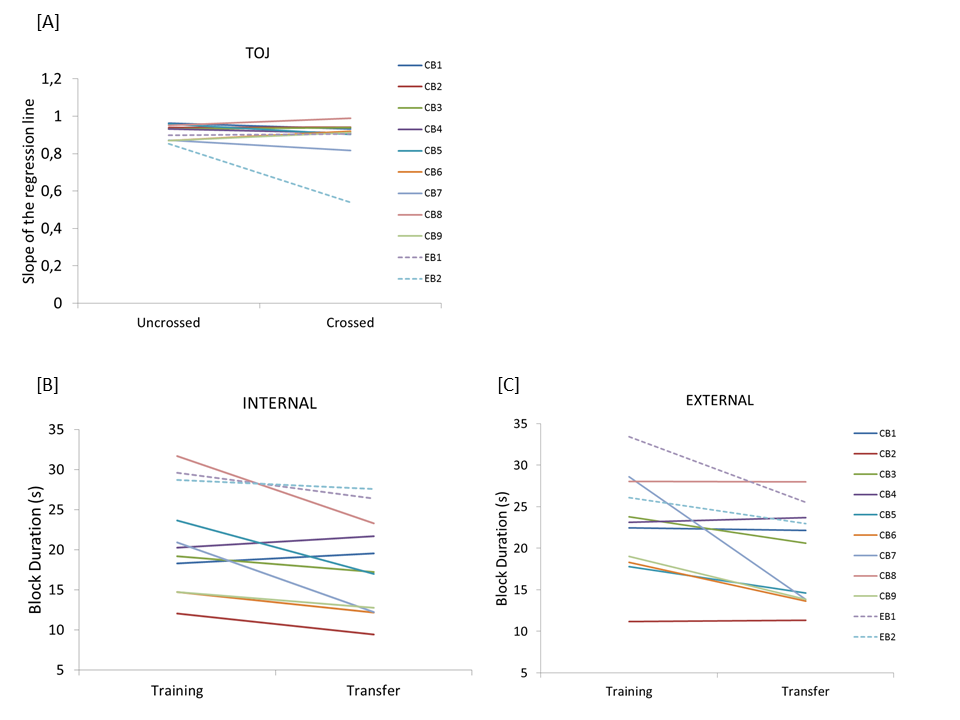


**Supplemental figure 1.** Individual data of the congenitally (CB) and early (EB) blind participants. [A] Value of the slopes of the regression lines in the TOJ task (in the uncrossed and crossed postures). The closer the slope is to 1, the better the performance. Transfer of motor sequence knowledge in the internal [B] and external [C] conditions of the motor sequence learning task. Improvement in performance is marked by a decrease of the duration (from the training to the transfer session) necessary to complete the different representations (internal vs. external) of the sequence.
